# Therapeutic interventions for osteoarthritis of the wrist: a systematic review and meta-analysis

**DOI:** 10.12688/f1000research.16218.2

**Published:** 2018-12-10

**Authors:** Benjamin Dean, Shwan Henari, Neal Thurley, Chris Little, Ian McNab, Nicholas Riley

**Affiliations:** 1 Nuffield Department of Orthopaedics, Rheumatology and Musculoskeletal Sciences (NDORMS), University of Oxford, Oxford, Oxon, OX3 7LD, UK; 2 Nuffield Orthopaedic Centre, Oxford University Hospitals NHS Trust, Oxford, Oxon, OX3 7LD, UK; 3 Bodleian Health Care Libraries, University of Oxford, Oxford, Oxon, OX3 9DU, UK

**Keywords:** wrist, osteoarthritis, ulnocarpal, radiocarpal, TFCC, review, intervention, surgery

## Abstract

**Background:** In order to evaluate the effectiveness of interventions for osteoarthritis of the wrist in adults we performed a systematic review and meta-analysis.

**Methods: **The MEDLINE and EMBASE via OVID, CINAHL and SPORTDiscus via EBSCO databases were searched from inception to 25 ^th^ April  2018.All randomised controlled clinical trials (RCTs) and any prospective studies of adults with wrist osteoarthritis investigating any intervention with a comparator were included.  Data were extracted and checked for accuracy and completeness by pairs of reviewers. Primary outcomes were pain and function. Comparative treatment effects were analysed by random effects at all time points.

**Results: **Three RCTs were identified for inclusion after screening and all had a high risk of bias. Two compared proximal row carpectomy (PRC) with four corner fusion (4CF) for post-traumatic osteoarthritis, while the other compared leather with commercial wrist splints in patients with chronic wrist pain, of which a small group had wrist osteoarthritis.

**Conclusion: **There is no prospective study comparing operative to non-operative treatment for wrist osteoarthritis, while there is a paucity of prospective studies assessing the effectiveness of both non-operative and operative interventions.  Further research is necessary in order to better define which patients benefit from which specific interventions.

**Registration:** The review protocol was registered with PROSPERO under the registration number CRD42018094799.

## Introduction

Osteoarthritis of the wrist is a diverse and poorly understood clinical condition, likely relating to the complexity of the anatomy and biomechanics of the human wrist joint ^1^. Wrist pain is a relatively common clinical problem, accounting for an annual consultation prevalence rate of 58 in 10,000 patients in the UK ^2^, which is around one-tenth the rate of consultation for back pain, the most common site of musculoskeletal pain. The prevalence of radiographic wrist osteoarthritis varies widely in the literature ^3– 5^, while there is a lack of epidemiological research relating pain to structural change in this condition.

Different anatomical areas of the wrist may be affected by osteoarthritis; the radiocarpal, the distal radioulnar, the ulnocarpal, the midcarpal (including the scaphotrapeziotrapzoid (STT)) and the pisotriquetral joint ^6^. Further complexity is added by the variety of terms used to describe ulnocarpal osteoarthritis, such as ulnocarpal abutment and ulnocarpal impaction syndrome, while tears and/or degeneration of the triangular fibrocartilage complex (TFCC) are intimately involved in ulnocarpal osteoarthritis and distal radioulnar joint synovitis ^7^. Osteoarthritis of the wrist may also occur after trauma, such as scaphoid non-union advanced collapse (SNAC), while the pattern of osteoarthritis seen in scapholunate advanced collapse (SLAC) may be post-traumatic or degenerative ^8^. 

Non-operative treatment includes activity modification, education, analgesia, physiotherapy, splintage and corticosteroid injections ^9^. Operative treatment is reserved for patients who fail non-operative measures, the specifics of which are determined by the anatomical pattern of the osteoarthritis ^10^. A wide array of operations are carried out on the wrist for osteoarthritis. Wrist arthroscopy has been increasingly performed in recent years, having both diagnostic and therapeutic components, including debridement and TFCC repair ^11^. Other procedures include denervation, excision arthroplasty, partial and total arthroplasty, partial and total wrist fusion and osteotomies. There is a scarcity of published material relating to the number of elective surgical procedures carried out on the wrist. Melamed *et al.* have described trends in the US relating to total wrist fusion and wrist arthroplasty ^12^. Jain *et al*. estimated that the number of wrist arthroscopies carried out per annum in the USA was approximately 25,000 in 2006, while the interest in and the number of surgeons performing wrist arthroscopy has been on the rise in recent years ^11^. 

Our aim was to perform a systematic review of the effectiveness of available interventions for osteoarthritis of the wrist in terms of patient-reported outcome measures and to assess the rates of adverse outcomes associated with these interventions.

## Methods

The systematic review was developed in accordance with the PRISMA statement, using methodology described in the Cochrane Handbook for Systematic Reviews of Interventions. The protocol was developed prospectively and peer reviewed locally before registration on the PROSPERO database ( CRD42018094799).

### Data sources and searches

A comprehensive search strategy was created in collaboration with a research librarian (N.T.) and was designed to capture all relevant articles pertaining to inventions for osteoarthritis of the wrist ( Supplementary Material 1). The full search strategy is detailed on the PROSPERO website. The search strategy was applied to the following bibliographic databases from database inception until 24th April 2018: MEDLINE and EMBASE via OVID, CINAHL and SPORTDiscus via EBSCO.

### Inclusion/exclusion criteria

The inclusion and exclusion criteria were defined prospectively during the protocol stage. All prospective studies relating to interventions for osteoarthritis of the wrist were included. Studies had to contain an intervention and a comparator (i.e. both non-randomised controlled trials, and randomised controlled trials, including semi/quasi randomised, cluster randomised trials and prospective comparative case series). Any therapeutic intervention or control treatments were included. We included ulnocarpal, distal radioulnar, radiocarpal, piso-triquetral, scaphotrapeziotrapezoid and midcarpal osteoarthritis. For the purposes of this review we have included all ulnocarpal disorders, such as ulnar abutment, ulnar impaction syndrome and triangular fibrocartilage complex degeneration, which is debatable but arguably fits within the most widely used definition of ‘osteoarthritis’ ^13^. Post-traumatic arthritis was also included (SLAC and SNAC). We excluded studies involving children and adolescents (age <18 years), and studies relating to inflammatory arthritis such as rheumatoid arthritis or psoriatic arthritis. Only studies published in a peer-reviewed English-language journal were included.

### Selection of studies

Duplicates were removed and relevant studies identified from the search were imported into Covidence for screening. Studies were independently screened by title and abstract by two authors (B.J.F.D. and S.H.). This was followed by a full-text evaluation of the selected studies from the first selection step by these authors. Disagreement between the two reviewers was resolved by consensus involving a third author (N.R.)

### Data extraction

Two reviewers (S.H. and B.J.F.D) independently extracted data. Data was extracted using a custom data extraction sheet in Covidence. Any inconsistencies between the two reviewers’ forms were resolved by consensus discussion. A third review (N.R.) was available for any disagreement that could not be resolved by this initial discussion.

If data was not available from full-text articles or trial registrations, authors were contacted to provide this information. If authors were not contactable for additional data, then this aspect of the study was excluded from the data synthesis. If contactable authors did not respond to initial requests, they were sent two subsequent reminders over a minimum of 6 weeks. If there was still no response for the additional data, then this aspect of the study was excluded from the data synthesis.

### Risk of bias assessment

Included studies were assessed for risk of bias by two independent raters (B.J.F.D. and S.H.) using the Cochrane Collaboration’s tool for assessing risk of bias in randomised trials ^14^. This followed the description in the Cochrane Handbook for Systematic Review of Interventions, version 5.1 (Part 2: 8.5.1) ^14^. Any disagreements between ratings were resolved by discussion between the raters. A third party (N.R.) was available in any case where disagreements persisted after discussion.

### Data analysis

Descriptive analysis was performed for all demographic, intervention and outcome data to facilitate narrative interpretation and comparison across studies. It was decided that a direct-comparison meta-analysis would only be performed if data was available for similar time-points, outcomes and interventions across two or more studies. As this was not possible with the identified studies, we conducted a narrative synthesis of the results based on the domains of interest.

## Results

### Studies identified

**Figure 1.**
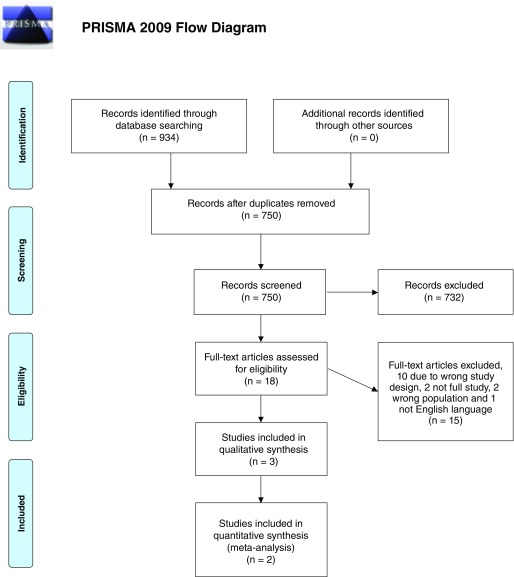
PRISMA flow diagram.

After duplicates were removed, 750 studies were identified by the search. After screening by full-text, three studies were identified as eligible for inclusion ( Figure 1). These were all randomized controlled trials (RCTs). The number of studies identified and excluded at each stage is detailed in Figure 1.

Study characteristics of the included trials including the interventions and comparators are provided in Table 1. Of the three included randomised controlled trials, two compared proximal row carpectomy with four corner fusion for post-traumatic osteoarthritis ^15, 16^, while the remaining RCT compared leather with commercial wrist splints in patients with chronic wrist pain, of which a small group had wrist osteoarthritis ^17^. Table 2 details the basic demographics of the intervention and comparator groups, as well as the details about the outcome data provided. The full details of all included studies and the forest plots are included within the Supplementary Material 3–7.

**Table 1.** Study characteristics.

Author	Year	Journal	Setting	Population	Study type	Intervention	Comparator	Primary outcome	Outcomes	Time points
*Aita* *et al. ^16^*	2016	Rev Bras Ortop	Hand surgery unit, secondary care	Adults with SNAC stage 2	RCT, parallel group	Proximal row carpectomy	Four corner fusion	DASH	VAS, Grip strength, range of movemment	Baseline and 74 month final follow up
*Bisneto* *et al. ^15^*	2011	Clinics	Orthopaedic surgery unit, secondary care	Adults with grade 1 or 2 SNAC/SLAC	RCT, parallel group	Proximal row carpectomy	Four corner fusion	DASH	VAS, Grip strength, range of movemment, Jebsen Taylor	Baseline, 3 months, 6 months and final 12 month follow up
*Thiele* *et al. ^17^*	2009	BMC Musc Dis	Occupational therapy department, secondary care	Chronic wrist pain	RCT, crossover	Leather splint	Commercial wrist splint	AUSCAN	COPM, Grip strength	Baseline 3 weeks and 6 weeks

SNAC, scaphoid non-union advanced collapse; RCT, randomised controlled trial; DASH, disabilities of arm, hand and shoulder; VAS, visual analogue score; SLAC, scapholunate advanced collapse; AUSCAN, Australian/Canadian Hand Osteoarthritis Index; COPM, Canadian Occupational Performance Measure.

**Table 2.** Details of study participants demographics, inclusion/exclusion criteria and whether data was provided.

Author	Year	Inclusion criteria	Exclusion criteria	Intervention group mean age, years (sd)	Comparator group mean age, years (sd)	Intervention group numbers/sex	Comparator group numbers/sex	Data comments
Aita *et al.* ^16^	2016	SNAC stage 2 and aged 18 to 60	Metabolic bone disease, bilateral problems and previous surgery	32.4 (9.4)	40.4 (8.9)	13/12M 1F	14/12M 2F	Full data provided
Bisneto *et al.* ^15^	2011	SNAC/SLAC grade 1 or 2	Inflammatory arthritis, midcarpal joint disease, failure to attend follow up	43.4 (10.1)	42 (10.6)	Not stated	Not stated	Incomplete data and author did not respond to multiple emails
Thiele *et al.* ^17^	2009	Chronic wrist pain, mixed population of osteoarthritis and inflammatory arthritis	Under 18 years of age, no functional impairment, seeking compensation, significant comorbidity	64 (range 18–82)		25/12M 13F		Data set for OA group not published and author did not respond to multiple emails

SNAC, scaphoid non-union advanced collapse; SLAC, scapholunate advanced collapse; OA, osteoarthritis.

The RCT by Aita *et al*. compared PRC (13 patients) with 4CF (14 patients) in adults with stage 2 SNAC. There were significant baseline differences between the two groups, the PRC group was significantly younger and had significantly greater range of movement pre-operatively. In terms of outcomes there was no observed difference in disabilities of arm, shoulder and hand (DASH), visual analogue score (VAS) pain and range of movement between PRC and 4CF, but a greater improvement in grip strength with PRC (SMD 0.61 95% CI 0.04-1.17) at final follow up. 

Bisneto *et al.* showed similar outcomes in terms of DASH and VAS pain in an RCT comparing PRC with 4CF in patients with a diagnosis of either post-traumatic SNAC or SLAC (10 patients in each group). The 4CF group did have significantly greater grip strength than the PRC group on the affected side pre-operatively and this persisted post-operatively. Unfortunately, it was not possible to obtain further data from the authors to enable a more detailed analysis.

Thiele *et al*. conducted a single-blind crossover RCT comparing the use of leather and commercial wrist splints for treating chronic wrist pain in adults ^17^. Of 25 included patients only six had the diagnosis of osteoarthritis, with the remaining 19 being inflammatory arthritis. Unfortunately, it was not possible to obtain the data pertaining to this osteoarthritis subgroup and further analysis was precluded. 

### Adverse events

Aita *et al*. reported one complication in each group ^16^. One patient in the PRC group developed symptomatic radiocarpal osteoarthritis requiring total wrist arthrodesis, while one patient in the 4CF group developed a ‘pseudoarthrosis’ (non union of the intercarpal fusions) which did not require further intervention. Bisneto *et al*. reported one complication in the 4CF group (‘reflex sympathetic dystrophy’) and five complications in the PRC group (two cases of ‘reflex sympathetic dystrophy’ and three cases synovitis; none of these complications required further surgical intervention ^15^.

### Risk of bias

**Figure 2.**
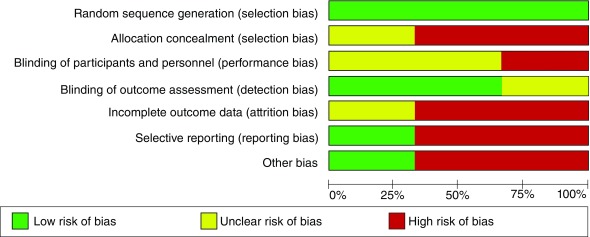
Risk of bias graph: review authors' judgements about each risk of bias item presented as percentages across all included studies.

**Figure 3.**
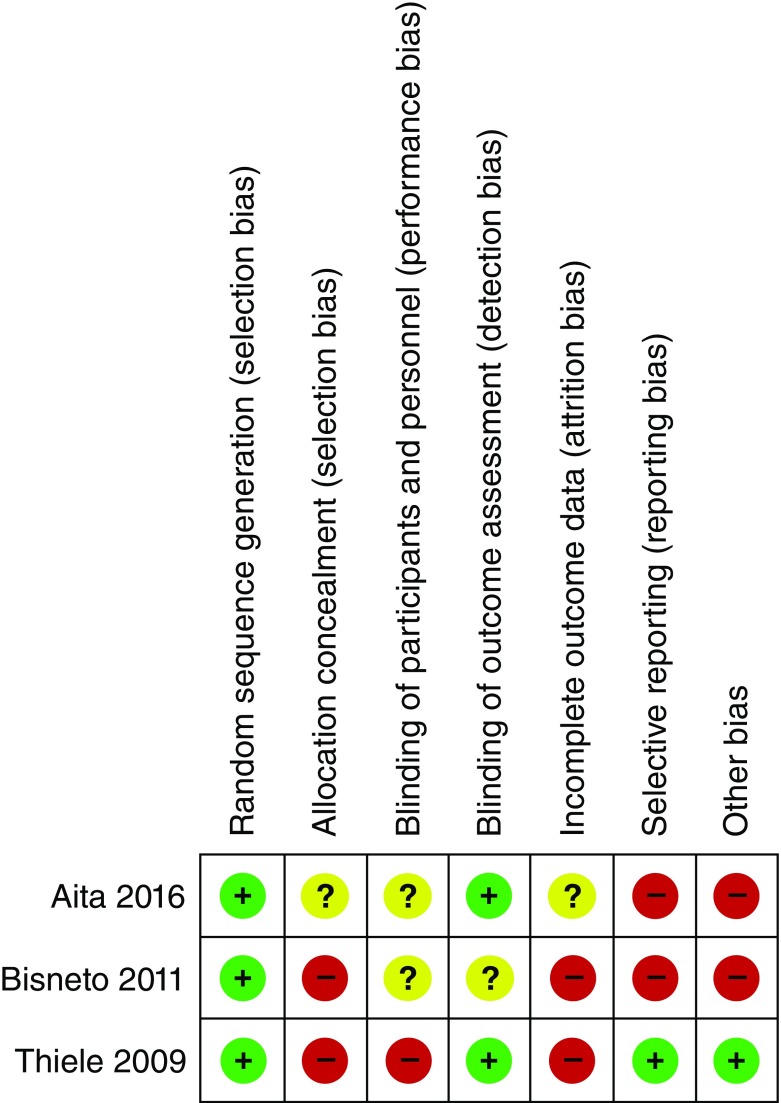
Risk of bias summary: review authors' judgements about each risk of bias item for each included study.

All criteria were judged as low, high or unclear risk of bias. Overall, all studies were deemed to be at a high risk of bias, particularly in terms of allocation concealment, incomplete outcome data and selecting reporting. Full risk of bias assessment is available in Figure 2 and Figure 3. Specifically the studies by Aita *et al*. and Bisneto *et al*. were both single-centre studies, did not specify a primary outcome measure and did not fully report outcome data. None of the three studies mentioned power calculations and all studies were relatively small in terms of participant numbers. All three studies used a random method of sequence generation. The studies by Aita *et al*. and Thiele *et al*. did blind the outcome assessors; however, the blinding of outcome assessment was not specified by Bisneto *et al*. Blinding of the participants was not possible in the study by Thiele *et al*., while it was not made clear in the studies by Aita *et al*. and Bisneto *et al*.

**Figure 4.**
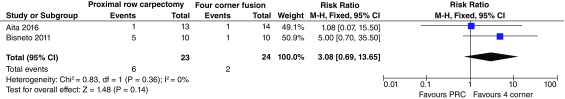
Forest plot of comparison: 1 Proximal row carpectomy vs Four corner fusion, outcome: adverse events.

***Meta analysis***. As a result of the degree of heterogeneity in terms of study interventions and the incomplete outcome data, it was determined that a meta-analysis of the outcomes was not possible. We carried out a meta-analysis of adverse events for 4CF vs PRC, as these were reported by two trials ^15, 16^ and the forest plot of this data is shown in Figure 4. No significant difference was noted in the risk of adverse events between these two groups (risk ratio 3.08 95% CI 0.69-13.65). It is important to note that the way in which ‘adverse events’ were defined by these two studies was unclear and may have been significantly different. This result should therefore be interpreted with caution.

Extracted study data, available as a RevMan 5 file RevMan 5 can be downloaded from: https://community.cochrane.org/help/tools-and-software/revman-5/revman-5-download. Click here for additional data file. Copyright: © 2018 Dean B et al. 2018 Data associated with the article are available under the terms of the Creative Commons Zero "No rights reserved" data waiver (CC0 1.0 Public domain dedication).

## Discussion

Based on this systematic review of the published literature, we have identified that there is no prospective study comparing operative to non-operative treatment for wrist osteoarthritis, while there is a paucity of prospective studies comparing the effectiveness of both non-operative and operative interventions. All three included studies were at high risk of bias, especially regarding reporting and blinding, and had other significant methodological weaknesses. In our opinion, the available evidence to inform treatment choices for this common clinical condition is surprisingly limited. 

The manner in which this lack of evidence results in a difficulty managing patients is saliently demonstrated by the example of the choice between PRC or 4CF for the treatment of SLAC or SNAC. Mulford *et al*. reviewed the literature in this area and remarked at the highly biased nature of the evidence base, the vast majority of which was retrospective case series ^18^. Of the three studies included in this review, two compared PRC with 4CF; however, the biased and small nature of these studies means that these studies do not substantially aid clinicians in making an evidence based decision in managing this group of patients.

The management of many other relatively common subtypes of wrist osteoarthritis is similarly hampered by a lack of high-quality evidence. This is demonstrated in the case of degenerative ulnocarpal disorders, which may also referred to as ulnar abutment, or ulnar impaction syndrome where the available evidence base consists exclusively of low-quality and highly biased retrospective case series, which invariably demonstrate no meaningful difference in outcome between different surgical interventions ^19– 22^. Not only are there no prospective studies, but to our knowledge no study has compared non-operative with operative intervention. This leaves both patients and clinicians with a dilemma in which the evidence base makes it very hard to decisively support any specific intervention.

The prevalence of radiographic wrist osteoarthritis varies widely in the literature. The seminal epidemiological study by Kellgren and Lawrence demonstrated a prevalence of radiographic wrist osteoarthritis of approximately 10% in men and 5% in women, notably this was in the 55 to 64 age bracket ^4^. Van Saase *et al*. demonstrated a prevalence of 9.1% and 12.4% in the 55–59 and the 60–64 year old age groups respectively ^5^. A much lower prevalence was reported in the Framingham study of between 1% and 2% in a group of mean age 58.9 years ^3^. Katayama reported a prevalence of radiographic ulnar sided wrist osteoarthritis in 12.8% of patients presenting to their orthopaedic wrist service with a mean age of 53.8 years; however, this study did not investigate the relationship between radiographic change and symptoms ^23^. There is also a paucity of high quality epidemiological evidence relating to the association between radiological changes and patient reported symptoms such as pain and dysfunction. The only studies which have investigated how radiological changes may relate to wrist pain have been conducted in young gymnastic populations ^24^. 

The absence of high quality evidence presents a challenge to clinicians treating wrist osteoarthritis. Although wrist osteoarthritis is more heterogeneous and less common than osteoarthritis affecting other joints such as the hip and knee, this should not mean that prospective evidence is perpetually absent. Whether in the form of prospective case series, cohort studies or randomised clinical trials, it is clear that higher-quality evidence is needed to guide practice in the management of wrist osteoarthritis. This may relate to simple non-operative interventions such as splintage, all the way up to the more invasive procedures such as total wrist arthroplasty and total wrist arthrodesis. **

## Conclusions

There is no prospective study comparing operative and non-operative treatment for wrist osteoarthritis, and an overall paucity of prospective studies comparing the effectiveness of both non-operative and operative interventions. Further research is necessary in order to better define which patients benefit from which specific interventions.

## Data availability

The data referenced by this article are under copyright with the following copyright statement: Copyright: © 2018 Dean B et al.

Data associated with the article are available under the terms of the Creative Commons Zero "No rights reserved" data waiver (CC0 1.0 Public domain dedication). 

**Dataset 1. Extracted study data, available as a RevMan 5 file.** RevMan 5 can be downloaded from: https://community.cochrane.org/help/tools-and-software/revman-5/revman-5-download. DOI: https://doi.org/10.5256/f1000research.16218.d217625 ^25^.

## Supplementary material

Supplementary Material 1. Full search histories.

.

Supplementary Material 2. Completed PRISMA checklist.

.

Supplementary Material 3. Forest plot of Aita *et al.* ^16^ DASH in PRC versus four corner fusion.

.

Supplementary Material 4. Forest plot of Aita *et al.* ^16^ VAS in PRC versus four corner.

.

Supplementary Material 5. Forest plot of Aita *et al.* ^16^ Grip strength in PRC versus four corner fusion.

.

Supplementary Material 6. Forest plot of Aita *et al.* ^16^ ROM wrist in PRC versus four corner fusion.
