# Supplementary material for: Therapeutic interventions for osteoarthritis of the wrist: a systematic review and meta-analysis
Source: F1000Res. 2018 Dec 10;7:1484. Originally published 2018 Sep 18. [Version 2] doi: 10.12688/f1000research.16218.2 (PMC6281017; doi:10.12688/f1000research.16218.2)
Supplement: Supplementary file 2 [file f1000research-7-19026-s0001.tgz › 85da5bf4-d4ce-48ad-be55-8f9d12ba040d_supp_1_wrist_osteoarthritis_search_strategies.pdf]

Database: Ovid MEDLINE(R) Epub Ahead of Print, In-Process & Other Non-Indexed Citations, Ovid MEDLINE(R) Daily and Ovid MEDLINE(R) <1946 to Present>
Search Strategy: (25/04/18)

----

1 Osteoarthritis/ (33574)
2 Arthritis/ (34242)
3 arthriti*.ti,ab. (160524)
4 1 or 2 or 3 (201315)
5 Wrist/ (8155)
6 wrist*.ti,ab. (33256)
7 Wrist Joint/ (9119)
8 ulnocarpal.ti,ab. (266)
9 distal radioulnar.ti,ab. (1280)
10 radiocarpal.ti,ab. (1336)
11 pisotriquetral.ti,ab. (97)
12 piso-triquetral.ti,ab. (17)
13 scaphotrapez*.ti,ab. (258)
14 scapho-trapez*.ti,ab. (92)
15 trapeziometacarpal.ti,ab. (691)
16 trapezio-metacarpal.ti,ab. (78)
17 midcarpal.ti,ab. (555)
18 mid-carpal.ti,ab. (64)
19 radiolunate.ti,ab. (304)
20 radio-lunate.ti,ab. (44)
21 radioscaphoid.ti,ab. (187)
22 radio-scaphoid.ti,ab. (24)
23 triscaphe.ti,ab. (18)
24 capitolunate.ti,ab. (99)
25 triquetrohamate.ti,ab. (30)
26 capitohamate.ti,ab. (21)
27 (ulnar* adj3 abutment*).ti,ab. (18)
28 (ulnar* adj3 imping*).ti,ab. (47)
29 (ulnar* adj3 impaction*).ti,ab. (166)
30 scapholunate advanced collapse.ti,ab. (152)
31 scapho-lunate advanced collapse.ti,ab. (6)
32 scaphoid non-union advanced collapse.ti,ab. (21)
33 scaphoid nonunion advanced collapse.ti,ab. (79)
34 5 or 6 or 7 or 8 or 9 or 10 or 11 or 12 or 13 or 14 or 15 or 16 or 17 or 18 or 19 or 20 or 21 or 22 or 23 or 24 or 25 or 26 (40526)
35 4 and 34 (4935)

36 27 or 28 or 29 or 30 or 31 or 32 or 33 or 35 (5222)

37 randomized controlled trial.pt. (459451)

38 controlled clinical trial.pt. (92359)

39 randomized.ab. (410017)

40 placebo.ab. (188534)

41 drug therapy.fs. (2012152)

42 randomly.ab. (289405)

43 trial.ab. (426191)

44 groups.ab. (1789105)

45 37 or 38 or 39 or 40 or 41 or 42 or 43 or 44 (4191406)

46 exp animals/ not humans.sh. (4449585)

47 45 not 46 (3622777)

48 36 and 47 (1005)

49 *Arthritis, Rheumatoid/ (74398)

50 rheumatoid.ti. (65418)

51 ra.ti. (2571)

52 *Arthritis, Psoriatic/ (3922)

53 psoria*.ti. (29330)

54 49 or 50 or 51 or 52 or 53 (116155)

55 48 not 54 (481)

****

Database: Embase <1974 to 2018 April 24>

Search Strategy: (25/04/18)

----

1 exp *arthritis/ (276001)

2 exp arthritis/ (423505)

3 arthriti*.ti,ab. (229912)

4 1 or 3 (357127)

5 2 or 3 (453948)

6 *wrist/ (6900)

7 wrist/ (24989)

8 wrist*.ti,ab. (44072)

9 ulnocarpal.ti,ab. (319)

10 distal radioulnar.ti,ab. (1337)

11 radiocarpal.ti,ab. (1543)

12 pisotriquetral.ti,ab. (96)

13 piso-triquetral.ti,ab. (21)

14 scaphotrapez*.ti,ab. (275)

15 scapho-trapez*.ti,ab. (99)
16 trapeziometacarpal.ti,ab. (752)
17 trapezio-metacarpal.ti,ab. (84)
18 midcarpal.ti,ab. (608)
19 mid-carpal.ti,ab. (72)
20 radiolunate.ti,ab. (349)
21 radio-lunate.ti,ab. (53)
22 radioscaphoid.ti,ab. (213)
23 radio-scaphoid.ti,ab. (35)
24 triscaphe.ti,ab. (22)
25 capitolunate.ti,ab. (115)
26 triquetrohamate.ti,ab. (28)
27 capitohamate.ti,ab. (23)
28 (ulnar* adj3 abutment*).ti,ab. (28)
29 (ulnar* adj3 imping*).ti,ab. (61)
30 (ulnar* adj3 impaction*).ti,ab. (177)
31 scapholunate advanced collapse.ti,ab. (168)
32 scapho-lunate advanced collapse.ti,ab. (8)
33 scaphoid nonunion advanced collapse.ti,ab. (77)
34 scaphoid non-union advanced collapse.ti,ab. (23)
35 6 or 8 or 9 or 10 or 11 or 12 or 13 or 14 or 15 or 16 or 17 or 18 or 19 or 20 or 21 or 22 or 23 or 24 or 25 or 26 or 27 (47965)
36 7 or 8 or 9 or 10 or 11 or 12 or 13 or 14 or 15 or 16 or 17 or 18 or 19 or 20 or 21 or 22 or 23 or 24 or 25 or 26 or 27 (51233)
37 4 and 35 (7255)
38 5 and 36 (8767)
39 28 or 29 or 30 or 31 or 32 or 33 or 34 or 37 (7587)
40 28 or 29 or 30 or 31 or 32 or 33 or 34 or 38 (9054)
41 randomized controlled trial/ (500107)
42 single blind procedure/ or double blind procedure/ (178726)
43 crossover procedure/ (55253)
44 random*.tw. (1296595)
45 (random or ((singl* or doubl*) adj (blind* or mask*)) or crossover or cross over or factorial* or latin square or assign* or allocat* or volunteer*).ti,ab. (1168791)
46 41 or 42 or 43 or 44 or 45 (1948286)
47 (exp animals/ or nonhuman/) not human/ (6423821)
48 46 not 47 (1698419)
49 39 and 48 (602)
50 40 and 48 (669)
51 *rheumatoid arthritis/ (106757)

52 rheumatoid.ti. (89226)

53 ra.ti. (6564)

54 *psoriatic arthritis/ (9043)

55 psoria*.ti. (41434)

56 51 or 52 or 53 or 54 or 55 (160861)

57 50 not 56 (306)

****

****

COCHRANE LIBRARY

Search Name: wrist osteoarthritis

Last Saved: 25/04/2018 14:21:36.870

Description:

ID	Search
#1	MeSH descriptor: [Osteoarthritis] this term only
#2	MeSH descriptor: [Arthritis] this term only
#3	arthriti*
#4	#1 or #2 or #3
#5	MeSH descriptor: [Wrist] this term only
#6	wrist*
#7	MeSH descriptor: [Wrist Joint] this term only
#8	ulnocarpal
#9	"distal radioulnar"
#10	radiocarpal
#11	pisotriquetral
#12	piso-triquetral
#13	scaphotrapez*
#14	scapho-trapez*
#15	trapeziometacarpal
#16	trapezio-metacarpal
#17	midcarpal
#18	mid-carpal
#19	radiolunate
#20	radio-lunate
#21	radioscaphoid
#22	radio-scaphoid

#23 triscaphe

#24 capitolunate

#25 triquetrohamate

#26 capitohamate

#27 ulnar* near/3 abutment*

#28 ulnar* near/3 imping*

#29 ulnar* near/3 impaction*

#30 scapholunate advanced collapse

#31 scapho-lunate advanced collapse

#32 scaphoid non-union advanced collapse

#33 scaphoid nonunion advanced collapse

#34 #5 or #6 or #7 or #8 or #9 or #10 or #11 or #12 or #13 or #14 or #15 or #16 or #17 or #18 or #19 or #20 or #21 or #22 or #23 or #24 or #25 or #26

#35 #4 and #34

#36 #27 or #28 or #29 or #30 or #31 or #32 or #33 or #35

#37 rheumatoid:ti

#38 ra:ti

#39 psoria*:ti

#40 #37 or #38 or #39

#41 #36 not #40
