# Supplementary figures and images for: Therapeutic interventions for osteoarthritis of the wrist: a systematic review and meta-analysis
Source: F1000Res. 2018 Dec 10;7:1484. Originally published 2018 Sep 18. [Version 2] doi: 10.12688/f1000research.16218.2 (PMC6281017; doi:10.12688/f1000research.16218.2)

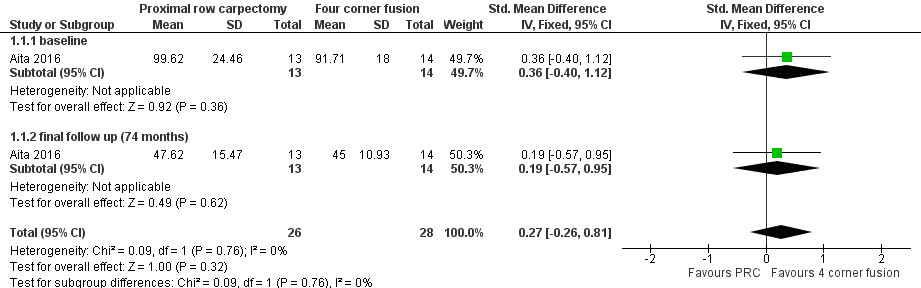

Supplement: Supplementary file 4 [file f1000research-7-19026-s0003.tgz › 8277515a-f1ae-4e33-b9b9-432d3986f6b7_supp_3_Forest_plot.png]

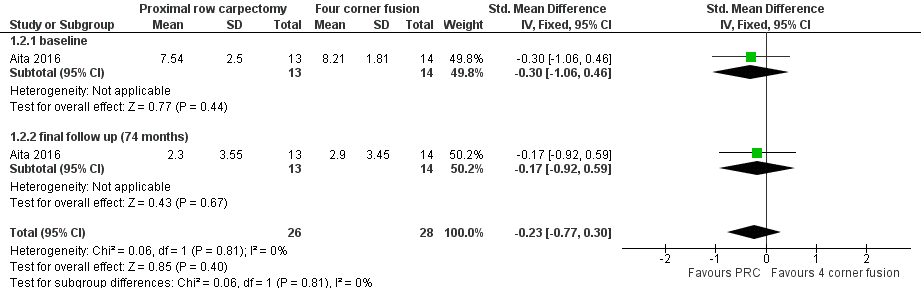

Supplement: Supplementary file 5 [file f1000research-7-19026-s0004.tgz › 91bdfc56-f87e-4f8d-8902-a30b014c843a_supp_4_Forest_plot.png]

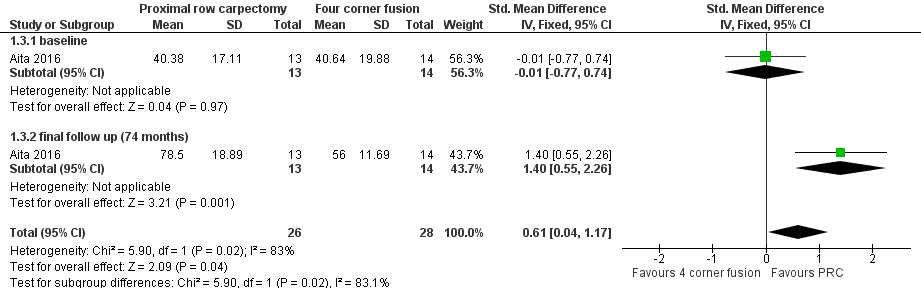

Supplement: Supplementary file 6 [file f1000research-7-19026-s0005.tgz › 49bcdb25-e81e-4ffa-968e-ae4529547f78_supp_5_Forest_plot.png]

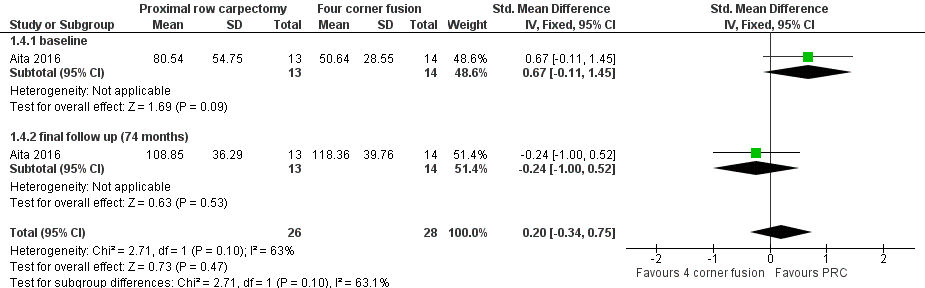

Supplement: Supplementary file 7 [file f1000research-7-19026-s0006.tgz › 25ddda9e-7c73-422a-ae11-48257f9633e7_supp_6_Forest_plot.png]
