# Supplementary material for: Therapeutic interventions for osteoarthritis of the wrist: a systematic review and meta-analysis
Source: F1000Res. 2018 Dec 10;7:1484. Originally published 2018 Sep 18. [Version 2] doi: 10.12688/f1000research.16218.2 (PMC6281017; doi:10.12688/f1000research.16218.2)
Supplement: Peer review history

# Peer review: Referee response for version 1

**DOI:** 10.5256/f1000research.17709.r40811

**Published:** 2018-12-03

**Authors:** Benjamin E. Smith, Katie Smith

**Affiliations:** 1 Derby Hospitals NHS Foundation Trust, Derby, UK; 2 Physiotherapy Department, London Road Community Hospital, Derby Hospitals NHS Foundation Trust, Derby, UK; 3 University of Nottingham, Notttingham, UK

This is an interesting, well written, and well conducted systematic review highlighting the lack of prospective studies comparing operative and non-operative treatment for wrist osteoarthritis, and lack of evidence evaluating the effectiveness of any intervention for osteoarthritis of the wrist.

 The introduction and method section is clear and appropriate, and the interpretation of the results seems suitable, given the lack of evidence and papers found.

 Reviewing, and cross checking the PROSPERO, the search terms used seem suitable for the inclusion criteria specified.

 There appears an error on the Exclusion: age >18 years in PROSPERO. Should read ‘age < 18’.

 I assume no attempt to search for unpublished data?

 Are there any numbers and percentages for agreement/disagreements for inclusion/extraction/scoring?

 Only a discussion point. If ‘there is a lack of epidemiological research relating pain to structural change in this condition’, as mentioned in the introduction, is there an argument to be calling it ‘wrist pain’, rather than ‘wrist OA’?

We have read this submission. We believe that we have an appropriate level of expertise to confirm that it is of an acceptable scientific standard.

# Response

**Published:** 2018-12-03

**Authors:** Benjamin Dean

**Affiliations:** University of Oxford, UK

Many thanks for taking the time to review the manuscript.

We have changed the erroneous '>' on Prospero to '<'.

In terms of review agreement - this is not recorded on Covidence unfortunately.

For the purposes of this review it was appropriate to use the term 'wrist osteoarthritis' as the specific scope of the review was to assess the evidence related to surgical intervention for this specific type of structural abnormality. Given the pack of evidence as regards the diagnosis and treatment of common wrist conditions, it is certainly worth considering how one should label these conditions in the future, both in the clinical and research settings. A review focused at 'wrist pain' instead of 'wrist osteoarthritis' would encompass a wider range of clinical conditions including diseases such as inflammatory arthritis, 'non-specific' wrist pain and tendinopathy, and this was not our aim.

# Peer review: Referee response for version 1

**DOI:** 10.5256/f1000research.17709.r40020

**Published:** 2018-12-03

**Authors:** Kirsty Challen

**Affiliations:** 1 Emergency Department, Lancashire Teaching Hospitals NHS Foundation Trust, Preston, UK

This is a well presented systematic review of the published literature on operative and non-operative interventions for wrist osteoarthritis. It could be strengthened with inclusion of grey literature (eg. searches of clinicaltrials.gov).

 I am not sure that it was appropriate to carry out a meta-analysis of adverse outcomes, as these seem to have been defined differently between the studies.

I have read this submission. I believe that I have an appropriate level of expertise to confirm that it is of an acceptable scientific standard.

# Response

**Published:** 2018-12-03

**Authors:** Benjamin Dean

**Affiliations:** University of Oxford, UK

Many thanks for taking the time to review our manuscript.

 The point regarding the meta-analysis is valid and we shall update the manuscript to include a description of this limitation.

# Peer review: Referee response for version 1

**DOI:** 10.5256/f1000research.17709.r38438

**Published:** 2018-10-08

**Authors:** Anju Jaggi

**Affiliations:** 1 Royal National Orthopaedic Hospital (RNOH), Stanmore, UK

This is a well presented systematic review paper representing selection criteria, data for analysis, risk of bias very well diagrammatically.

 The interpretation and conclusion is clear and justified in light of the results, there is a lack of evidence for both patients and clinicians to decide if operative or non-operative interventions are best for osteoarthritis of the wrist being reasonable how important this is in the context of osteoarthritis in general. 

 I would accept this paper for publication and have no amendments to make however I do not have appropriate statistical knowledge to fully recommend the forest plot data in the study.

I have read this submission. I believe that I have an appropriate level of expertise to confirm that it is of an acceptable scientific standard.
